# Synergistic effect of phytochemicals on cholesterol metabolism and lipid accumulation in HepG2 cells

**DOI:** 10.1186/s12906-018-2189-6

**Published:** 2018-04-05

**Authors:** Ennian Leng, Yuan Xiao, Zhentao Mo, Yiqi Li, Yueyue Zhang, Xiaosi Deng, Min Zhou, Chaochao Zhou, Zengxuan He, Jingyi He, Lu Xiao, Junming Li, Wenna Li

**Affiliations:** 1 Zhuhai Campus, Zunyi Medical University, Jinwan District, Zhuhai, 519041 Guangdong China; 2 Pharmaceutical Preparation Section, Guizhou Province People’s Hospital, Guiyang, Guizhou China

**Keywords:** Crocin, Chlorogenic acid, Geniposide, Quercetin, Synergistic interaction, Lipid accumulation

## Abstract

**Background:**

Crocin (CRO), chlorogenic acid (CGA), geniposide (GEN), and quercetin (QUE) are all natural compounds with anti-obesity properties, in particular, hypolipidemic effects, which have been widely used for the treatment of obesity-related metabolic diseases. However, it is not yet known whether these compounds interact synergistically. Here, we investigated the effects and molecular mechanisms of CRO, CGA, GEN, QUE, and a combination of all four compounds (CCGQ), on lipid accumulation in human hepatoma (HepG2 cells).

**Methods:**

The optimal concentration of CRO, CGA, GEN, QUE to stimulate HepG2 cells proliferation was determined using MTT assay. HepG2 cells were pretreated with 10 μmol/L simvastatin, 1 μmol/L CRO, 30 μmol/L CGA, 10 μmol/L GEN, 10 μmol/L QUE, and CCGQ (a combination of 1 μmol/L CRO, 30 μmol/L CGA, 10 μmol/L GEN, and 10 μmol/L QUE) for 24 or 48 h. Oil red O staining and extracellular TC and TG levels were detected. The RT-PCR was used to observe on cholesterol metabolism-related gene expression. Immunocytochemistry and western-blot assayed the 3-hydroxy-3-methylglutaryl-coenzyme (HMGCR) protein expression in HepG2 cells.

**Results:**

Compared to those of control, we demonstrated that treating HepG2 cells for 48 h with CCGQ resulted in a strong synergistic effect, causing a marked decrease in lipid deposition in comparison to individual treatments, in both triglyceride and total cholesterol (CRO, 5.74- and 1.49-folds; CGA, 3.38- and 1.12-folds; GEN, 4.04- and 1.44-folds; QUE, 3.36- and 1.24-folds; simvastatin, 5.49- and 1.83-folds; and CCGQ, 7.75- and 2.20-folds), and Oil red O staining assays. In addition, CCGQ treatment increased ATP-binding cassette transporter (ABCA1), cholesterol 7α-hydroxylase (CYP7A1), and AMP-activated protein kinase 2α (AMPKα2) mRNA expression, while decreasing sterol regulatory element binding protein 2 (SREBP2), and liver X receptor alpha (LXRα) mRNA expression. Notably, CCGQ was more effective in decreasing HMGCR expression than the individual treatments.

**Conclusion:**

The CCGQ combination has potential, both as a complementary therapy for hyperlipemia, and in preventing further obesity-related complications.

## Background

Obesity has become a global epidemic which is strongly associated with many metabolic diseases, such as coronary heart disease, type 2 diabetes mellitus, and non-alcoholic fatty liver disease [1, 2]. Effective long-term solutions to reduce obesity have remained elusive until now. Many obesity-related metabolic abnormalities are characterized by dyslipidemia, in particular the formation of excessive lipid deposits in non-adipose tissue, such as the liver. These deposits cause lipotoxicity, resulting in tissue damage, fibrosis and potential organ failure [3]. There have been numerous investigations into the role of dyslipidemia in the pathophysiology of obesity [4, 5]. Decreasing hyperlipemia is therefore considered a protective strategy to treat obesity-induced diseases [6, 7].

In the last decade, many studies have focused on a variety of natural products, including botanical extracts or phytochemicals, for the prevention and treatment of obesity. In these studies, phenolic compounds and carotenoids have attracted a great deal of interest. Phenolic compounds, such as phenolic acids and flavonoids, have pharmacological properties involved in the regulation of plasma lipids, and are increasingly being studied [8]. Crocin (CRO) is the only water-soluble carotenoid in nature, and has been shown to be effective in reducing diet-induced obesity in animals, as well as improving the blood lipid profile [9, 10]. Chlorogenic acid (CGA), ubiquitously found in plants, is a phenolic acid, which has been shown to exert anti-obesity effects and improve lipid metabolism [11]. It has been reported that hypocholesterolemic effects are the primary benefit of CGA treatment [12]. Geniposide (GEN), an iridoid glycoside, has also recently been demonstrated to infer protective effects against hyperlipemia and obesity in rats [13]. Quercetin (QUE), commonly found in glycosidic form in plants, is the most abundant flavonoid in the typical human diet, and exerts anti-obesity activity through a range of molecular pathways [14, 15].

*Gardenia jasminoides Ellis* (gardenia) fruit, of the Rubiaceae family, was reported to decrease body weight, fat percentage, and BMI, lower high insulin, GLP-1, and leptin levels, and reduce insulin resistance in middle-aged obese women. It has been reported that CRO, CGA, rutin, and GEN are the main components of gardenia fruit [16]. The anti-obesity effect of gardenia fruit may be attributed to synergistic interactions between its various phytochemical constituents, and analyzing the activity of these active components is a key step to understanding this effect and underlying mechanisms. It is therefore necessary to study the impact of the combination of these compounds on dyslipidemia, but no study has so far focused on this. Rutin is the most common, and important, glycosidic form of QUE [15] and cellular models can clarify how these phytochemicals interfere with lipid metabolism to prevent obesity at the molecular level. This study therefore aimed to investigate hypolipidemic effects and molecular mechanisms of combined therapy with CRO, CGA, GEN, and QUE in human hepatoma HepG2 cells.

## Methods

### Chemicals

**Fig. 1:**
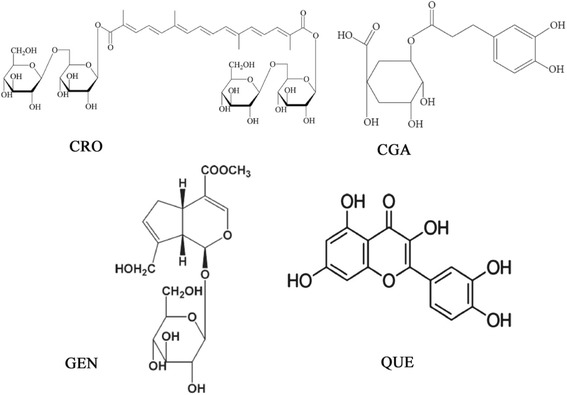
Chemical structures of CRO, CGA, GEN and QUE

CGA, GEN and QUE (purity > 98%, as determined by HPLC) were purchased from the National Institute for the Control of Pharmaceutical and Biological Products (Beijing, China). CRO was extracted and purified as previously reported (purity > 98%, as determined by HPLC) [17]. All drugs were dissolved in 0.1% dimethyl sulfoxide (DMSO). The chemical structures of purified CRO, CGA, GEN, and QUE are illustrated in Fig. 1. All other chemicals used were of molecular biology and analytical grade.

### Cell culture

The HepG2 human hepatoma cell line was purchased from the China Center for Type Culture Collection. HepG2 cells were grown and maintained in RPMI-1640 medium (Invitrogen, Waltham, MA) supplemented with 10% fetal bovine serum, 100 U/mL penicillin, and 100 μg/mL streptomycin at 37 °C in a humidified atmosphere of 5% CO_2_. Cells were subcultured upon reaching approximately 80–90% confluency.

### Cell viability assay

HepG2 cell growth was determined using the MTT assay. Cells (2 × 10^4^) were seeded into 96 well micro-titer plates containing 100 μL complete culture medium per well, and incubated for 24 h at 37 °C. Cultures were transferred into serum-free medium 12 h prior to performing the assay. A range of concentrations of CRO (0.01, 0.1, 1, 10, and 100 μmol/L), CGA (0.3, 3, 30, 300, and 3000 μmol/L), GEN (0.1, 1, 10, 100, and 1000 μmol/L), and QUE (1, 10, 20, 40, 60, 80, and 100 μmol/L) were added. Cells were incubated for a further 24 or 48 h in an incubator (37 °C at 5% CO_2_), and then treated with MTT for 4 h. DMSO (150 μL) was added to each well, and plates were then shaken for 10 min. Absorbance was measured at 490 nm using a microplate reader (Bio-Rad). Cell viability in each group was presented as a percentage of the control.

### Oil red O staining and determination of extracellular TC and TG levels

HepG2 cells (2 × 10^4^) were seeded into 6- or 24-well plates and cultured. After incubation in the presence or absence of the different drugs for 48 h, cells were fixed with 4% polyformaldehyde, and then incubated in Oil red O working solution for 30 min. Microscopic images were taken to visualize red oil droplets with an Olympus microscope (Olympus Corporation, Tokyo, Japan). After incubation in the presence or absence of the different drugs for either 24 or 48 h, cell supernatant was used for analysis of total cholesterol (TC) and triacylglycerol (TG) levels. TC and TG kits (Beihuakangtai, Beijing, China) were used according to the manufacturer’s instructions.

### RT-PCR

Expression of the following genes was studied using semi-quantitative RT-PCR: ATP-binding cassette transporter (ABCA1), sterol regulatory element binding protein 2 (SREBP2), cholesterol 7α-hydroxylase (CYP7A1), liver X receptor alpha (LXRα), AMP-activated protein kinase 2α (AMPKα2) and 3-hydroxy-3-methylglutaryl-coenzyme A reductase (HMGCR). Primer pairs (Sangon, Shanghai, China) are listed in Table 1. RNA isolation (Total RNA Extractor, Sangon, Shanghai, China), cDNA synthesis (TaKaRa PrimeScript™ RT reagent Kit, Baosheng, Dalian, China), and amplification (TaKaRa Ex Taq PCR Kit, Baosheng, Dalian, China) were performed as per manufacturer’s instructions. Amplification was performed using a three step temperature cycle as follows: ABCA1, AMPKα2, and HMGCR: 32 cycles at 95 °C for 20 s, 57 °C for 30 s, and 72 °C for 45 s; SREBP2, LXRα, and glyceraldehyde-3-phosphate dehydrogenase (GAPDH): 32 cycles at 95 °C for 20 s, 56 °C for 30 s, and 72 °C for 45 s; and CYP7A1: 32 cycles at 95 °C for 20 s, 58 °C for 30 s and 72 °C for 30 s. Polymerization was performed at 72 °C for 5 min. Amplified product was analyzed in 1% agarose gel, photographed, and analyzed using Quality One Software (SPSS Inc., Chicago, IL). Each relative densitometric value was calculated after normalization to GAPDH.

**Table 1:** The primers of gene amplification

GENE	Sense	Anti-sense	Product
ABCA1	5’-AGGCCCAGACCTGTAAATGC-3’	5’-CTCACCAACCTTGCCAACTTC-3’	450 bp
SREBP2	5’-CAGACGCCAAGATGCACAAG-3’	5’-TGGCTCATCTTTGACCTTTGC-3’	323 bp
CYP7A1	5’-CATTTGGGCACAGAAGCATTG-3’	5’-AGGCAGCGGTCTTTGAGTTAG-3’	174 bp
NR1H3 (LXR α)	5’-AAGAAACTGAAGCGGCAAGA-3’	5’-AGCAATGAGCAAGGCAAACT-3’	555 bp
PRKAA2 (AMPK α2)	5’-TCAATCGTTCTGTCGCCAC-3’	5’-ATACGGTTTGCTCTGACTTCG-3’	530 bp
HMGCR	5’-CTCCGCAGGCTATTTGTTCAG-3’	5’-GCTAAGAGCGTTCGTGGGTC-3’	620 bp
GAPDH	5’-ACTCCTCCACCTTTGACGCTG-3’	5’-CTCTCTTCCTCTTGTGCTCTTGC-3’	182 bp

### Immunocytochemistry

HepG2 cells were plated into 6-well chamber slides. After treatment with the different drugs for 48 h, the slides were fixed in 4% neutral buffered paraformaldehyde for 30 min at room temperature, subsequent HMGCR detection. Immunostaining was carried out using a kit (Boaoseng, Beijing, China, SP-0023). The endogenous peroxidase was inactivated by treated the slides with 0.3% hydrogen peroxide for 10 min. After washed with PBS, the slides were added into normal goat serum to incubate for 10 min at 37 °C. Rabbit polyclonal HMGCR antibody (1:50) (Boaoseng, Beijing, China, LOT. YE0905W, bs-5068R) was used as the primary antibody to incubated cells at 4 °C for overnight after washed. The slides were washed and incubated with biotinylated secondary antibody for 30 min. And then slides were added into peroxidase-labeled streptavidin for another 30 min. Slides were stained with 3,3′-diaminobenzidine for 25 min. Cells were incubated with nonimmune serum or PBS to control antibody specificity, and then the nuclei were counterstained with H&E.

### Western blot

Total cell protein from each sample was extracted using a kit (Protease inhibitor cocktail set I, Calbiochem, USA) and quantified using a Bio-Rad protein kit (Pierce, Rockford, IL). Equal quantities of protein were separated using 10% SDS-PAGE and transferred to PVDF membrane (Millipore Corporation, USA) overnight at 4 °C: the membrane was then blocked in 5% non-fat milk. The membrane was probed separately with the primary antibodies for GAPDH-HRP (1:1000, Kangcheng, Shanghai, China) and rabbit antihuman HMGCR (1:1000, Cell Signaling), and secondary antibodies (1:10000, Santa Cruz). Signal was detected using an ECL Kit (Millipore, USA) and developed on X-ray film. Protein bands were detected by ChemiDoc XRS systems and Quality One 4.6.1 (Bio-Rad, Hercules, CA) software, and GAPDH antibody was used as the internal control.

### Statistical analysis

All data were presented as means ± SEM, obtained from at least three separate experiments, and one representative image was selected for presentation in the figures. One-way analysis of variance followed by Dunnett’s test was used for comparison among groups (SPSS computer software version 13.0). *P* < 0.05 was considered significant.

## Results

### Effects of CRO, CGA, GEN, and QUE on HepG2 cell viability

**Fig. 2:**
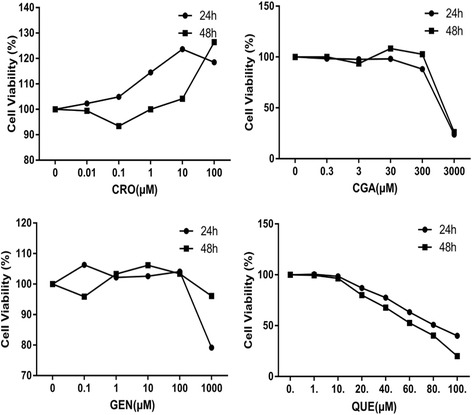
The effects of different concentrations of CRO, CGA, GEN and QUE on the viability of HepG2 cells for 24 h or 48 h. Data are expressed as mean ± SEM from at least five independent experiments

We examined the cytotoxicity of CRO, CGA, GEN, and QUE to determine suitable concentrations for the treatment of HepG2 cells. Results showed that 100 μM CRO, CGA, and GEN, and ≤ 10 μM QUE had no inhibitory effect on HepG2 cell viability after 24 or 48 h of treatment (Fig. 2). Therefore, subsequent experiments used the following concentrations: 10 μmol/L SIM, 1 μmol/L CRO, 30 μmol/L CGA, 10 μmol/L GEN, 10 μmol/L QUE, and a combination of 1 μmol/L CRO, 30 μmol/L CGA, 10 μmol/L GEN, and 10 μmol/L QUE (CCGQ), consistent with concentrations used in previous studies [18–21].

### CRO, CGA, GEN, QUE, and CCGQ inhibit lipid accumulation in HepG2 cells

**Fig. 3:**
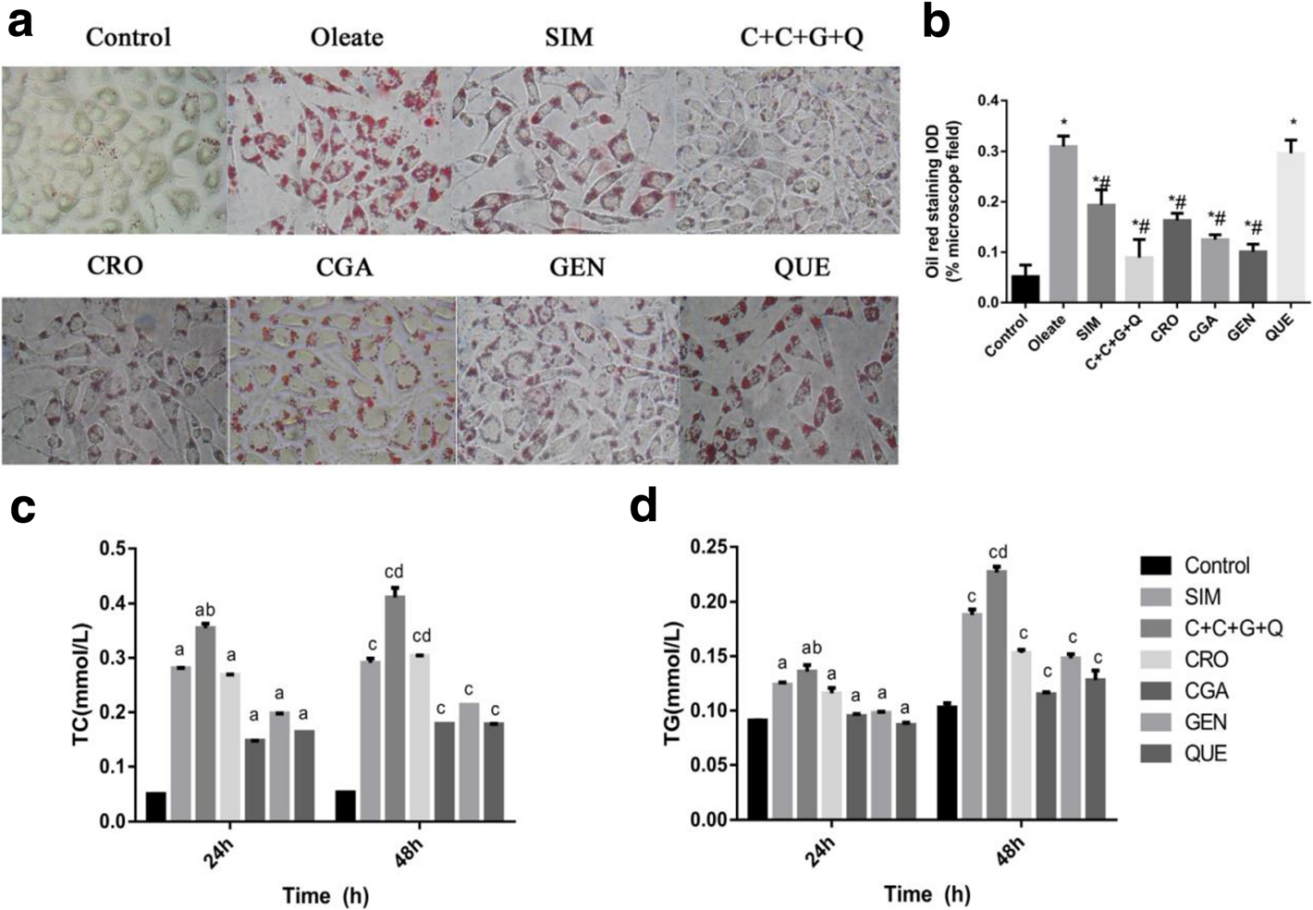
The effects of CRO, CGA, GEN, QUE and C + C + G + Q on lipid content in HepG2 cells. HepG2 cells were treated with Oleate (0.1 mmol/L), SIM (10 μmol/L), CRO (1 μmol/L), CGA (30 μmol/L), GEN (10 μmol/L), QUE (10 μmol/L) and C + C + G + Q (1 μmol/L + 30 μmol/L + 10 μmol/L + 10 μmol/L) for 24 h or 48 h. **a** After 48 h incubation with different drugs, the images of cells were observed by microscope at 250 × original magnification showing lipid accumulation in cells stained by Oil red O. **b** The comparison of integral optical density (IOD) for Oil red staining in cells. Effects of different drugs on the secretion of (**c**) TC and (**d**) TG were observed by HepG2 cells in minimum essential medium. Data are expressed as mean ± SEM from at least four independent experiments. *P < 0.05 vs. control, ^#^*P* < 0.05 vs. Oleate. After 24 h, ^a^*P* < 0.05 vs. control, ^b^*P* < 0.05 vs. SIM. After 48 h, ^c^*P* < 0.05 vs. control, ^d^*P* < 0.05 vs. SIM

To test whether drugs affected lipid deposition, we examined lipid droplets in HepG2 cells using Oil red O staining. Compared with the control group, we found prominent lipid accumulation in the cytoplasm of HepG2 cells, using 0.1 mmol/L of oleate. Compared with the oleate group, treatment with SIM, CRO, CGA, GEN, and CCGQ significantly attenuated the accumulation of lipid droplets, however, QUE alone did not induce notable inhibition (Fig. 3a, b). To further assess lipid deposition, we determined TC and TG content in the culture medium of HepG2 cells (Fig. 3c, d). Consistent with Oil red O findings, all drugs elevated extracellular TC and TG levels after treatment for 24 or 48 h. CCGQ and CRO induced significant increases in concentrations of secreted TC in cells treated for 48 h, which were greater than effects produced by any other single drug. Compared with the SIM group, treatment with CCGQ for 24 or 48 h significantly increased extracellular TC and TG content (*p* < 0.05). These results indicate that combined treatment with CCGQ results in a much greater decrease in lipid accumulation in HepG2 cells than treatment with the drugs individually.

### Effect of CRO, CGA, GEN, QUE and CCGQ on cholesterol metabolism-related gene expression

**Fig. 4:**
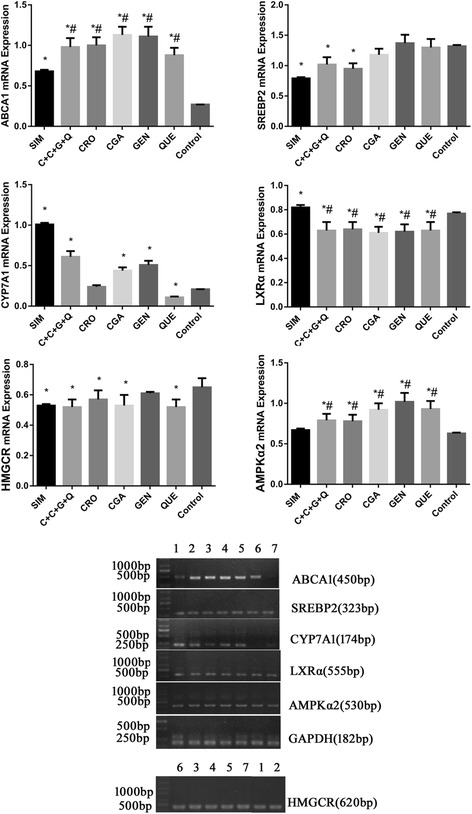
mRNA expression levels of the regulation cholesterol activities associated genes ABCA1, SREBP2, CYP7A1, LXRα, AMPKα2 and HMGCR in HepG2 cells. All genes were normalized with GAPDH. HepG2 cells were treated with (1) SIM (10 μmol/L), (2) C + C + G + Q (1 μmol/L + 30 μmol/L + 10 μmol/L + 10 μmol/L) (3) CRO (1 μmol/L), (4) CGA (30 μmol/L), (5) GEN (10 μmol/L), (6) QUE (10 μmol/L) and no drug for (7) Control respectively for 48 h. Data represent as mean ± SEM of three independent experiments. ^*^P < 0.05 vs. control, ^#^*P* < 0.05 vs. SIM

In order to further clarify the mechanism responsible for the changes in cholesterol levels of HepG2 cells, we next examined the mRNA expression levels of cholesterol metabolism-related enzymes (results shown in Fig. 4). All drugs increased ABCA1 mRNA expression in comparison with the control group, and had significantly greater effects than SIM treatment. CYP7A1 mRNA expression was significantly enhanced by treatment with SIM, CCGQ, CGA, and GEN, but was comparable to the control group in CRO-treated cells, and was lower than levels in all other groups in QUE-treated cells. All individual drugs except GEN significantly decreased HMGCR mRNA expression.

Numerous studies indicate that AMPK, LXRα and SREBP2 play key roles in regulating cholesterol homeostasis. As shown in Fig. 4, treatment with SIM, CCGQ, or CRO significantly inhibited SREBP2 expression compared to the control group, while the other drugs had no significant effect on SREBP2 expression. Treatment with SIM significantly up-regulated LXRα mRNA expression, while the opposite effect was observed in cells treated with all other drugs. All drugs except SIM enhanced AMPK2α expression compared with the control group.

### CRO, CGA, GEN, QUE, and CCGQ suppress HMGCR protein expression in HepG2 cells

**Fig. 5:**
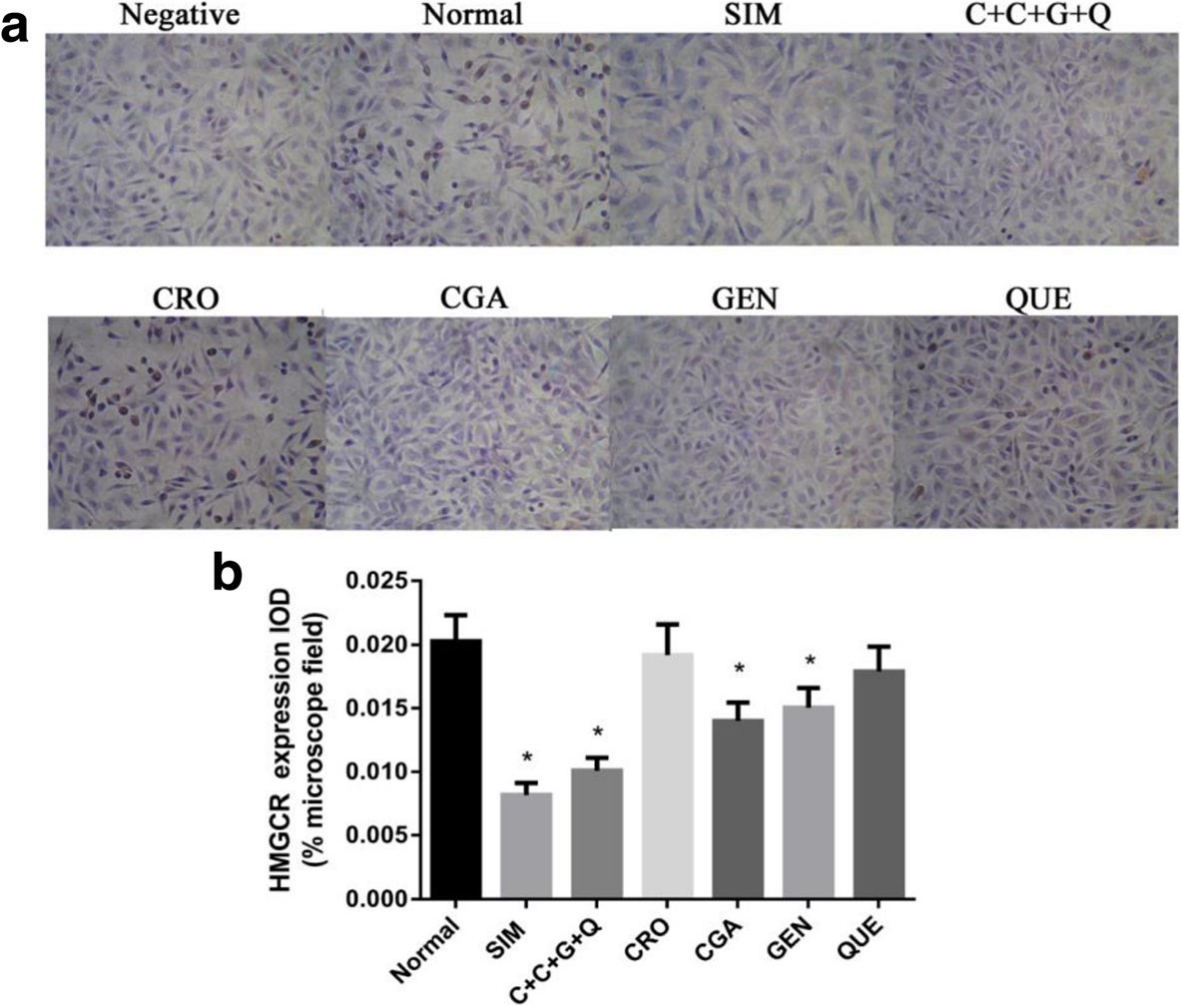
Expression of HMGCR in HepG2 cells. **a** Representative immunocytochemical images of HMGCR expression in cells. HepG2 cells were treated with SIM (10 μmol/L), C + C + G + Q (1 μmol/L + 30 μmol/L + 10 μmol/L + 10 μmol/L), CRO (1 μmol/L), CGA (30 μmol/L), GEN (10 μmol/L) and QUE (10 μmol/L) for 48 h respectively, and treated with PBS instead of the primary antibody of HMGCR for negative control, and with no drug for normal control. The brown granules in the cytoplasm represent HMGCR positive staining results. **b** The bar chart showing the comparison of the IOD for HMGCR expression in cells. Data represent as mean ± SEM of three independent experiments. *P < 0.05 vs. control, ^#^*P* < 0.05 vs. SIM

**Fig. 6:**
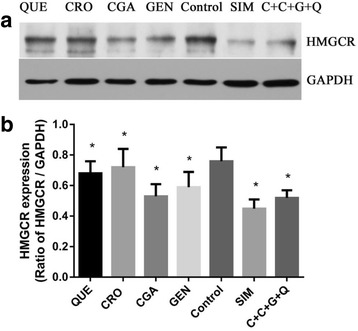
All drugs and their combination suppress HMGCR protein expression in HepG2 cells. **a** Western blotting analysis of HMGCR, (**b**) The densitonmetric scanning of HMGCR after normalization with GAPDH. HepG2 cells from 3 set of each group were pooled separately and homogenates was prepared for western blotting analysis of HMGCR. HepG2 cells were treated with QUE (10 μmol/L), CRO (1 μmol/L), CGA (30 μmol/L), GEN (10 μmol/L), SIM (10 μmol/L) and C + C + G + Q (1 μmol/L + 30 μmol/L + 10 μmol/L + 10 μmol/L) for 48 h respectively. Data represent as mean ± SEM of three independent experiments. *P < 0.05 vs. control

We also examined intracellular localization and expression of HMGCR protein by immunostaining and western blotting, after treatment for 48 h with the different drugs. Figure 5 demonstrates prominent accumulation of brown HMGCR granules localized in the cytoplasm of control, CRO, and QUE cells. Treatment with SIM, CGA, GEN, or CCGQ markedly reduced HMGCR expression compared with untreated cells. Consistent with these results, as shown in Fig. 6, HMGCR protein expression decreased in all treatment groups compared to the control group, but not as much as the SIM group. In particular, CCGQ combined treatment resulted in a much greater decrease in HMGCR expression than individual treatments.

## Discussion and conclusion

CRO, CGA, GEN and QUE are known to possess anti-obesity properties, in particular hypolipidemic effects, and have been widely used for treatment of obesity-related metabolic diseases [6, 7]. This is the first study to demonstrate that combined CCGQ treatment of HepG2 cells resulted in a marked decrease in lipid deposition in comparison to individual CRO, CGA, GEN, and QUE treatment, determined by Oil red O staining, and triglyceride and total cholesterol assays. In addition, with respect to the rate-limiting enzyme for cholesterol synthesis, pretreatment with CCGQ for 48 h had a potent decreasing effect on HMGCR compared to individual drugs, determined by immunohistochemistry and western-blot analysis, and induced genes related to cholesterol metabolism.

Similar to gardenia fruit, HPLC analysis showed that *Eucommia ulmoides* Oliver (eucommia) leaf mainly consists of CGA, in addition to GEN and QUE [22]. We previously demonstrated that CGA-enriched extract (CAEF) from eucommia leaf was more effective in lowering lipid levels than the same concentration of CGA, and deduced that the hypolipidemic effect of CAEF may be attributed to the synergistic effects of its other components [18]. We therefore selected CRO, CGA, GEN, and QUE for HepG2 cell treatment, to observe their synergistic lipid-decreasing effects. Neutral lipid drops were apparently decreased by all drugs except QUE: these results were further supported by the determination of TC and TG content in the supernatant of HepG2 cells, and combinations of these natural compounds were more effective than SIM or each individual compound. These positive outcomes may result from synergistic interactions between the different bioactive phytochemicals.

The liver plays a central role in cholesterol metabolism. Cholesterol homeostasis is governed by several genes, including HMGCR, CYP7A1, ABCA1, SREBPs, LXR, and AMPK. These genes maintain a complex network involving cholesterol uptake, synthesis, intracellular transport, and excretion [23, 24]. HMGCR is responsible for cholesterol synthesis [25], CYP7A1 catalyzes the conversion of cholesterol into bile acids, ABCA1 is involved in high-density lipoprotein (HDL) formation and facilitates the efflux of free cholesterol, SREBP2 positively regulates cholesterol biosynthesis genes, and activation of LXR is associated with increased lipogenesis and fat accumulation. HMGCR, CYP7A1, ABCA1, and SREBPs are all target genes of LXR [26–28]. AMPK, as an upstream kinase in lipid metabolism regulation, inhibits transcriptional activity of SREBPs [29], as well as ligand-induced LXR activity and production of endogenous LXR ligands [30]. The protective effects of CRO, CGA, QUE, GEN and their combination against hepatocytic cholesterol accumulation may therefore be studied by measuring changes in expression of these key hepatic transcription mRNAs.

Our data show that ABCA1, CYP7A1, and AMPK2α expression in HepG2 cells significantly increased in CCGQ-treated cells; however, HMGCR, SREBP2 and LXRα mRNA levels were significantly lower than those of the control group. Gene expression in the SIM group was similar to that of the CCGQ group, aside from up-regulated LXRα and a lack of increase in AMPK2α expression in SIM-treated cells. Again, similar effects were observed for ABCA1, AMPK2α, and LXRα mRNA expression in HepG2 cells, after treatment with CRO, CGA, QUE, and GEN. Nonetheless, we observed that CRO, CGA, and QUE also significantly decreased HMGCR mRNA expression, although this was not observed for the GEN group. Interestingly, out of all four phytochemicals, only CRO induced dramatically lower SREBP2 mRNA expression than the control group. On measuring the abundance of the CYP7A1 gene, responsible for the synthesis of bile acids, we observed significant differences between CRO-, CGA-, GEN-, and QUE-treated cells. CYP7A1 expression was repressed in the QUE group, CGA and GEN increased CYP7A1 expression, and no change was noted in the CRO group.

The role for QUE in suppressing LXRα gene expression and modulating lipid accumulation has previously been described in Huh7 cells [31]. The current study is the first demonstration that CRO, CGA, QUE, GEN, and CCGQ all significantly inhibited LXRα mRNA expression in HepG2 cells, in agreement with previous data from our group [18]. Although the expression of genes involved in cholesterol biosynthesis, bile acid synthesis, and cholesterol efflux is regulated by LXR, our data show that CCGQ up-regulated some genes involved in these processes (CYP7A1 and ABCA1) and down-regulated others (HMGCR and SREBP2). In recent reports, AMPK activation increased ABCA1 mRNA and protein expression, increasing the cholesterol efflux capacity of macrophages [32, 33]. Activation of AMPK by sulfated low molecular weight guluronate was linked to up-regulation of CYP7A1 in HepG2 cells [34]. Based on these studies, the up-regulation of CYP7A1 and ABCA1 may be attributed to AMPK activation by CCGQ. Further studies are required to fully understand the mechanism of AMPK activation.

In our study, HMGCR protein expression, measured by immunocytochemistry and western blot and confirmed by PCR, was inhibited to a greater extent in the CCGQ group than in cells treated with the individual drugs, suggesting that the four phytochemicals may be more efficient in combination than separately. These data are consistent with the reduction in hepatic lipid accumulation observed in Oil red O staining and lipid content analysis. Analysis of the relative expression of related genes, based on hepatic lipid concentration, also showed that CCGQ dramatically decreased hepatic lipid accumulation to a greater extent than the individual drugs.

Based on our observations, the preventative effect of CCGQ on lipid accumulation in HepG2 cells was considerably greater than that of SIM. Statins are the preferred drugs for treatment of hyperlipemia. However, evidence that they cause hazardous side effects, and are effective in only one-third of patients, highlights the need for alternative therapies [35].

The experimental findings of the present study primarily revealed that synergistic interactions between combined CRO, CGA, GEN, and QUE demonstrated positive effects against hepatic lipid accumulation. The anti-hyperlipidemic effect of these phytochemicals appears to be mediated by the modulation of AMPK, LXRα, and associated genes engaged in cholesterol synthesis, metabolism, and efflux. To our knowledge, this is the first report demonstrating the synergistic lipid-lowering actions of CRO, CGA, GEN and QUE. Although results from in vitro experiments cannot be directly extrapolated to anti-obesity and lipid-modulating clinical effects of gardenia fruit [16] and eucommia leaf [36], such studies will assist in elucidating the anti-hyperlipidemic mechanisms of their constituent compounds, either individually or in combination treatments. These results also suggest that combining active ingredients in traditional herbal medicine, to enhance their curative effects, may potentially improve hyperlipemia and further prevent obesity-related complications. However, more detailed studies are required to validate their synergistic interactions.

## Abbreviations

ABCA1: ATP-binding cassette transporter
AMPKα2: AMP-activated protein kinase 2α
CCGQ: The combination of CRO, CGA, GEN, and QUE
CGA: Chlorogenic acid
CRO: Crocin
CYP7A1: Cholesterol 7α-hydroxylase
DMSO: Dimethyl sulfoxide
GAPDH: Glyceraldehyde-3-phosphate dehydrogenase
GEN: Geniposide
HMGCR: 3-hydroxy-3-methylglutaryl-coenzyme
LXRα: Liver X receptor alpha
QUE: Quercetin
SIM: Simvastatin
SREBP2: Sterol regulatory element binding protein 2
TC: Total cholesterol
TG: Triacylglycerol
